# Directed PCR-free engineering of highly repetitive DNA sequences

**DOI:** 10.1186/1472-6750-11-87

**Published:** 2011-09-23

**Authors:** Annika Scior, Steffen Preissler, Miriam Koch, Elke Deuerling

**Affiliations:** 1 Molecular Microbiology, Department of Biology, University of Konstanz, 78457 Konstanz, Germany; 2 Konstanz Research School Chemical Biology, University of Konstanz, 78457 Konstanz, Germany

## Abstract

**Background:**

Highly repetitive nucleotide sequences are commonly found in nature e.g. in telomeres, microsatellite DNA, polyadenine (poly(A)) tails of eukaryotic messenger RNA as well as in several inherited human disorders linked to trinucleotide repeat expansions in the genome. Therefore, studying repetitive sequences is of biological, biotechnological and medical relevance. However, cloning of such repetitive DNA sequences is challenging because specific PCR-based amplification is hampered by the lack of unique primer binding sites resulting in unspecific products.

**Results:**

For the PCR-free generation of repetitive DNA sequences we used antiparallel oligonucleotides flanked by restriction sites of Type IIS endonucleases. The arrangement of recognition sites allowed for stepwise and seamless elongation of repetitive sequences. This facilitated the assembly of repetitive DNA segments and open reading frames encoding polypeptides with periodic amino acid sequences of any desired length. By this strategy we cloned a series of polyglutamine encoding sequences as well as highly repetitive polyadenine tracts. Such repetitive sequences can be used for diverse biotechnological applications. As an example, the polyglutamine sequences were expressed as His_6_-SUMO fusion proteins in *Escherichia coli *cells to study their aggregation behavior *in vitro*. The His_6_-SUMO moiety enabled affinity purification of the polyglutamine proteins, increased their solubility, and allowed controlled induction of the aggregation process. We successfully purified the fusions proteins and provide an example for their applicability in filter retardation assays.

**Conclusion:**

Our seamless cloning strategy is PCR-free and allows the directed and efficient generation of highly repetitive DNA sequences of defined lengths by simple standard cloning procedures.

## Background

Expansions of DNA repeat sequences are associated with many inherited neurodegenerative diseases [1-3]. One of the best-studied examples for a trinucleotide expansion disease is the neurological disorder Huntington's chorea, where the accumulation of CAG triplets within the first exon of the gene encoding the Huntingtin (Htt) protein leads to an elongated polyglutamine (Poly-Q) stretch in the polypeptide. It has been shown that more than 36 consecutive glutamine residues are pathogenic as they promote Htt aggregation into amyloid-like fibrils [4]. The translation product of the first exon of the *htt *gene was previously used to study the aggregation behavior of Poly-Q proteins *in vitro *[5]. In order to investigate the influence of the length of the Poly-Q stretch on aggregation kinetics, we wanted to clone reporter constructs containing defined numbers of glutamine residues. We developed a PCR-free cloning strategy allowing us to create repetitive DNA sequences encoding glutamine stretches of defined length. These sequences were used to generate improved constructs for filter retardation assays to study Huntingtin aggregation *in vitro*.

Several methods have been described for cloning long DNA repeat tracts. However, most of them include PCR-based amplification steps, generate imperfect repeats, or result in a pool of clones that differ in the number of repeats [6-13]. As a consequence, additional effort is required to identify and isolate clones with the desired length of nucleotide repeats. Therefore, we developed a simple multi-cycle cloning strategy using synthetic oligonucleotides and Type IIS restriction endonucleases to engineer highly repetitive DNA fragments. Our approach is PCR-free and generates exclusively clones carrying the desired length of repeat sequences in each step. Moreover, it is not only suitable to multiply the length of existing repetitive sequences but also to vary the number of inserted repeats in every elongation cycle. Finally, we can easily recombine constructs of the same or different repeat lengths to accelerate the construction of the desired number of repeats.

## Results and discussion

**Figure 1:**
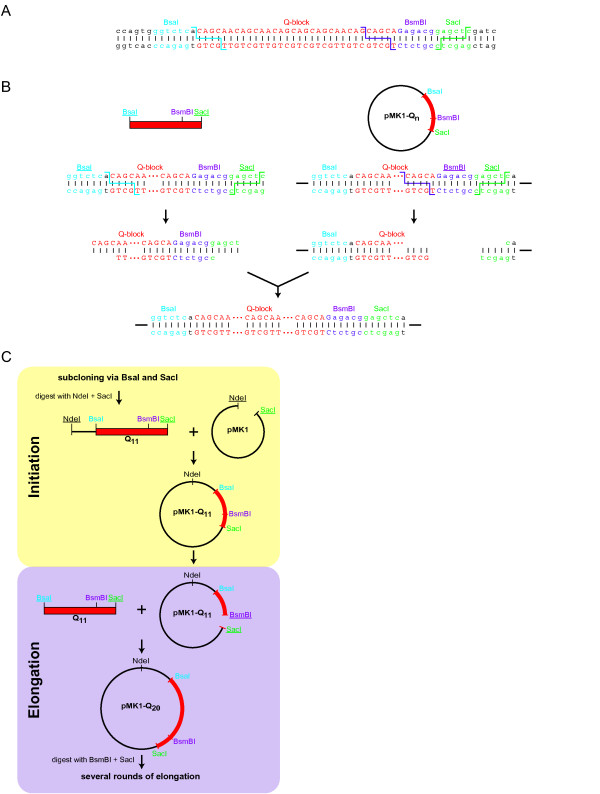
**Cloning strategy of Poly-Q encoding sequences**. (**A**) Annealed oligonucleotides used for cloning of the Poly-Q encoding regions. The recognition sequences and the corresponding cleavage sites of the restriction endonucleases are indicated by different colors. The orientation of the two inward facing Type IIS restriction sites (BsaI and BsmBI) results in cleavage within the Poly-Q encoding sequence and allows seamless elongation of the Poly-Q stretch. (**B**) Detailed representation of the elongation cycles. Digestion of the double-stranded oligonucleotides (BsaI/SacI) and the vector (BsmBI/SacI) resulted in compatible overhangs allowing for seamless elongation. Used restriction sites are underlined. (**C**) The annealed oligonucleotides were initially subcloned using BsaI and SacI and subsequently inserted into pMK1 via NdeI and SacI. For further elongation BsaI/SacI digested oligonucleotides were ligated into pMK1-Q_n _digested with BsmBI and SacI. Thereby the number of Q-encoding nucleotides was increased by nine and a new BsmBI site was introduced allowing subsequent rounds of elongation.

Type IIS restriction endonucleases cut DNA at defined distances outside their recognition site [14]. Thus, these enzymes allow the generation of single-stranded DNA overhangs of any desired sequence. The arrangement of inward facing Type IIS recognition sites at the ends of DNA fragments facilitates their fusion without incorporation of extraneous nucleotides. Therefore, such enzymes are widely used in DNA shuffling or seamless cloning strategies to construct synthetic genes and artificial protein polymers [15-19]. Based on this approach, we designed double-stranded oligonucleotides containing a defined number of central glutamine-encoding CAG and CAA triplets (Q-block) arranged in a non-regular fashion (Figure 1A). We decided on this approach because it has been shown previously that interruption of perfect CAG repeats by CAA triplets improves the stability of Poly-Q encoding sequences in *E. coli *[12]. The central triplet repeats were flanked by non-repetitive sequences that have two important functions: First, they ensure the specific annealing of the oligonucleotides, and secondly, they comprise two inward directed Type IIS restriction sites (BsaI and BsmBI) and a Type IIP restriction site (SacI). The Type IIS sites were designed to result in cuts within the repetitive sequence and to produce compatible DNA overhangs allowing for seamless elongation of the Poly-Q encoding sequence (Figure 1A and 1B). In our strategy, the BsaI and SacI restriction sites of the Q-block oligonucleotides are used to elongate a pre-existing Q-block on a plasmid digested with BsmBI and SacI (Figure 1C, lower panel). Importantly, the inward facing BsmBI site on the last introduced oligonucleotide remains intact upon ligation and is available for further insertions. Therefore, seamless elongation is based on the re-introduction of a unique Type IIS site in each elongation step (Figure 1B and 1C).

The antiparallel Q-block oligonucleotides described above were commercially synthesized, annealed, and subcloned via the BsaI and SacI restriction sites into a vector containing a unique BsaI site (Figure 1B and 1C). The following elongation steps of the Poly-Q encoding sequence only require the double-stranded oligonucleotides treated with the appropriate enzymes as described above and a cloning vector without BsmBI sites. Hence, we performed the elongation cycles in the plasmid pMK1 from which all BsmBI sites were removed by site-directed mutagenesis. The initial introduction of the subcloned Q-block fragment into pMK1 resulted in the plasmid pMK1-Q_11 _(Figure 1C, upper panel). As a consequence, this vector now contains a single BsmBI site downstream of the glutamine-encoding triplets, which could be used for the further elongation of the Q-block (Figure 1C). Next, pMK1-Q_11 _was digested with the enzymes BsmBI and SacI. The resulting cohesive ends were compatible with the overhangs of the double-stranded Q-block oligonucleotides cut by BsaI and SacI (Figure 1C). Ligation of the insert with pMK1-Q_11 _gave rise to pMK1-Q_20_. Thereby a unique BsmBI site was re-introduced downstream of the elongated Poly-Q encoding region (Figure 1C). As the cohesive ends of the annealed and digested oligonucleotides are only compatible with the doubly-cut plasmid, multiple insertions were efficiently prevented. Using this strategy the directed elongation of the Q-block was achieved by repeated cycles of digestion and ligation.

**Figure 2:**
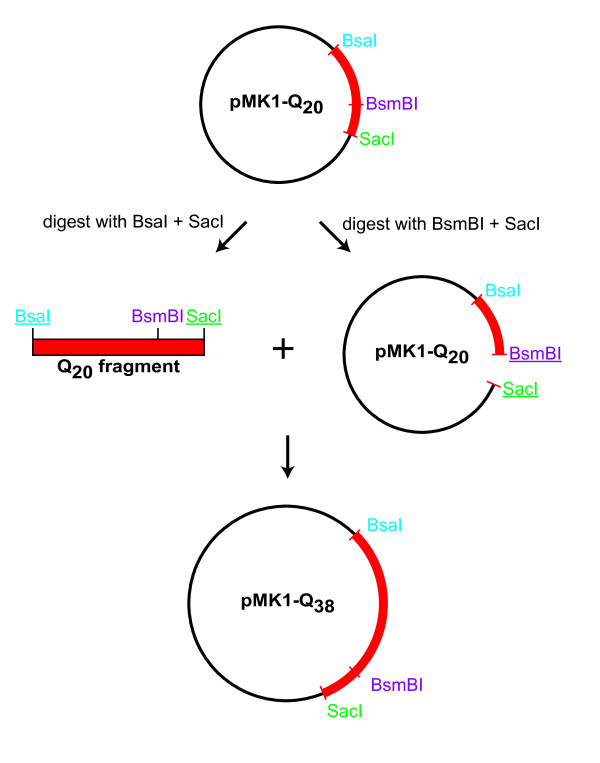
**Generation of long repetitive sequences can be accelerated**. The elongation of the Q-encoding region was accelerated by a simple modification of the cloning strategy. For example, the Poly-Q encoding region of the plasmid pMK1-Q_20 _can be excised with BsaI and SacI and ligated into the same vector digested with BsmBI/SacI. Thereby, the number of Q-encoding repeats can be almost doubled in a single cloning step.

**Figure 3:**
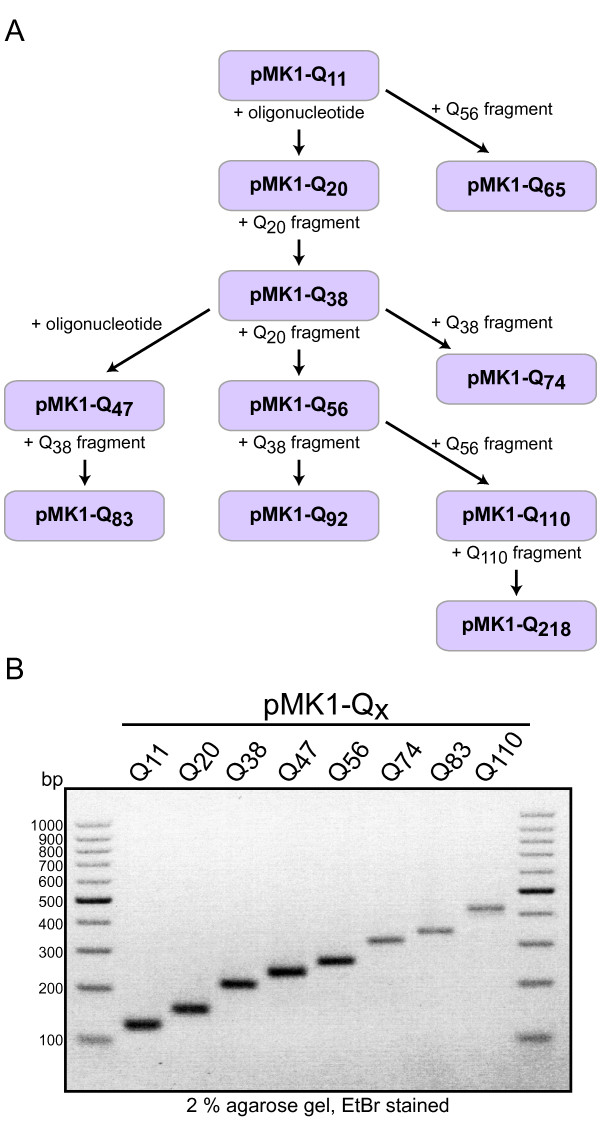
**Overview of the seamless cloning of repetitive sequences for the production of Poly-Q proteins**. (**A**) Genealogy of the pMK1-vectors. A pool of different pMK1 vectors was generated by elongation cycles using double-stranded oligonucleotides or Poly-Q encoding DNA fragments derived from pre-existing pMK1-Q_n _vectors. (**B**) Repetitive sequences of different lengths cloned by the described strategy in (A). pMK1-Q_n _plasmids encoding indicated Poly-Q stretches were digested with EcoRI (cuts 77 bp upstream of BsaI) and SacI. The inserts were separated on a 2% agarose gel and visualized by ethidium bromide (EtBr) staining.

Our approach has several additional advantages beyond providing a strategy for the directed elongation of repetitive DNA sequences. An important aspect is that the number of introduced nucleotides can be adjusted precisely in each round by defining the length of the Q-block of the synthetic oligonucleotides (Figure 1A). Furthermore, the generation of long repetitive sequences can be accelerated by simple modifications (Figure 2). For example, pMK1-Q_20 _was digested with BsaI and SacI and the resulting Q-block fragment was ligated with the pMK1-Q_20 _backbone cleaved with BsmBI and SacI. This slight adaptation of the protocol enabled the formation of pMK1-Q_38 _and demonstrates that the number of Q-encoding triplets could be rapidly increased in a single step. This allowed the fast generation of a pool of constructs with different lengths of the repetitive sequences that can be easily recombined (Figure 3A). Figure 3B shows the restriction digestion analysis of the constructed pMK1-Q_n _plasmids carrying inserts encoding the indicated Poly-Q stretches.

Our approach is also suitable to increase the length of repetitive sequences by multiple insertions as described previously [13]. Thereby, simultaneous digestion of the annealed oligonucleotides or pMK1-Q_n _plasmids with both Type IIS restriction enzymes would result in Q-block fragments with compatible overhangs (Figure 1A). This would allow for ligation of multiple Q-blocks in a single step.

**Figure 4:**
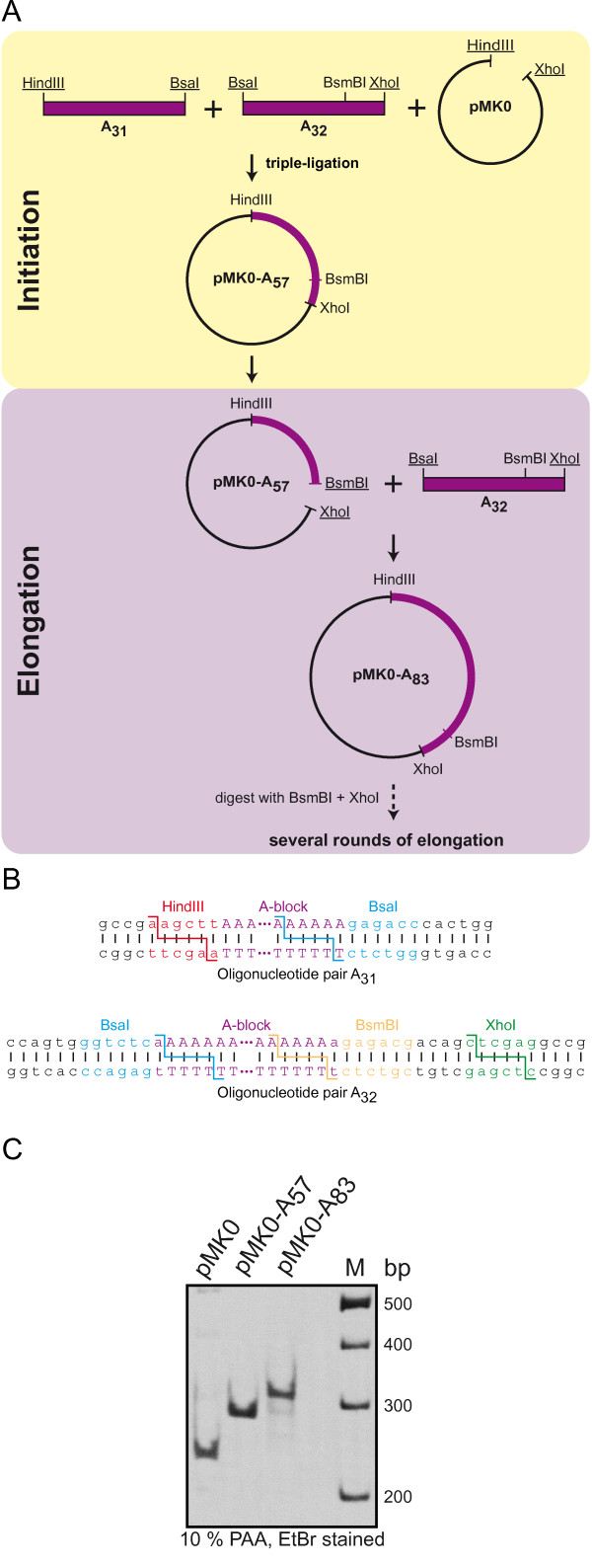
**Cloning of poly(A) constructs**. (**A**) Our basic strategy was also successfully applied to the cloning of adenine repeat sequences. Here two antiparallel oligonucleotide pairs that differ in their restriction sites and length of the poly(A) region, A_31 _and A_32_, were used for the initiation step. A_31_, A_32_, and pMK0 were digested with HindIII/BsaI, BsaI/XhoI, and HindIII/XhoI, respectively, and ligated in a single reaction to obtain pMK0-A_57_. For elongation the BsaI/XhoI digested oligonucleotide A_32 _was inserted into pMK0-A_57 _cut with BsmBI and XhoI. The elongation principle is the same as described above for the Poly-Q constructs. Used restriction sites are underlined. (**B**) Antiparallel oligonucleotide pairs used for cloning of the poly(A) sequences. The recognition sequences and the corresponding cleavage sites of the restriction endonucleases are indicated by different colors. The inward facing BsaI sites on A_31 _and A_32 _were designed in a way to allow the seamless fusion of the oligonucleotides via their A-blocks. (**C**) Initiation and elongation step of poly(A) cloning. pMK0-A_n _plasmids containing the indicated number of adenines or the empty pMK0 vector were digested with SacI (cuts 199 bp upstream of poly(A)) and XhoI. The inserts were separated on a 10% polyacrylamide (PAA) gel and visualized by ethidium bromide (EtBr) staining.

Importantly, our technique is not limited to generate Poly-Q encoding sequences but can also be used for other nucleotide repeats. One example of highly repetitive nucleotide sequences are the poly(A) tails attached to the 3' ends of eukaryotic mRNAs by poly(A)-polymerases. These poly(A) extensions are up to 250 nucleotides in length and they are important for mRNA stability and efficient translation initiation [20]. As polyadenylation is lacking in conventional *in vitro *transcription reactions, poly(A) fragments are usually fused to the 3' untranslated regions of the DNA templates. Nevertheless, controlling the length of the poly(A) sequence is difficult to achieve by conventional cloning strategies. We have successfully applied our novel approach for the directed elongation of repetitive DNA sequences to create poly(A) stretches of defined length in DNA templates for the *in vitro *production of polyadenylated mRNA (Figure 4). Therefore, we used A-block oligonucleotides instead of Q-blocks and applied the same basic strategy as described above. In this case we used two oligonucleotide pairs (A_31 _and A_32_) to generate a stretch of 57 adenines and an inward facing BsmBI site in a single initiation step (Figure 4A and 4B). The newly introduced BsmBI site was used to elongate the poly(A) region with the oligonucleotide pair A_32 _(Figure 4A, lower panel). A restriction digestion analysis of the initiation and elongation steps of poly(A) cloning is shown in Figure 4C.

**Figure 5:**
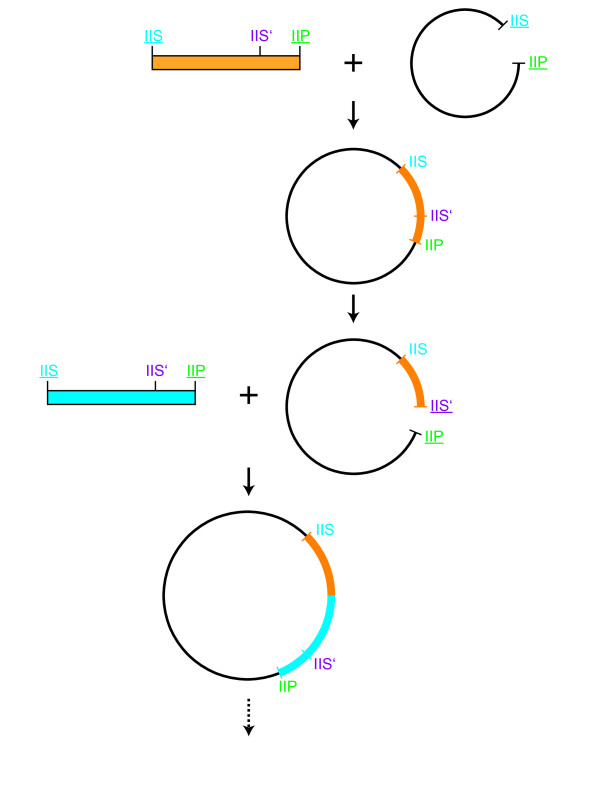
**Strategy to combine different repetitive sequences in a modular fashion**. Antiparallel oligonucleotide pairs with different repetitive sequences (orange and blue) can be fused on one plasmid if their Type IIS recognition sites (IIS) are designed in a way that they create compatible overhangs. Moreover, our strategy is not restricted to specific endonucleases. The use of any combination of one Type IIP (IIP) and two different compatible Type IIS enzymes is possible. Upon insertion of the first annealed and digested oligonucleotide pair (orange) via IIS and IIP, the resulting vector can be digested with IIS' and IIP to allow the incorporation of a second double-stranded oligonucleotide pair (blue).

Theoretically, the basic strategy can also be expanded to establish a modular system of double-stranded DNA oligonucleotides or PCR-amplified fragments carrying different sequences that can be fused in a directed fashion and in any desired order. This is possible as long as the recognition sites of the Type IIS enzymes are designed to produce compatible ends (Figure 5). Moreover, other combinations of Type IIS and Type IIP restriction endonucleases can be used instead of the ones chosen in this study.

**Figure 6:**
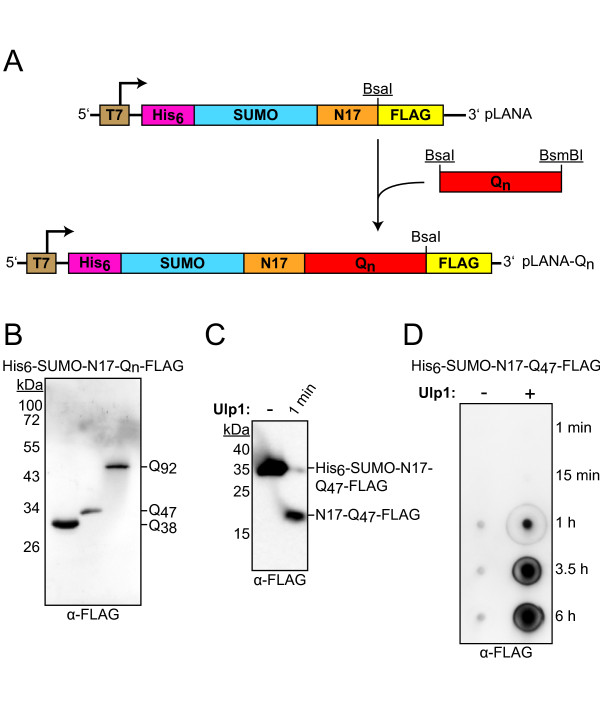
**Cloning and analysis of the final Poly-Q constructs**. (**A**) The final constructs encode an N-terminal His_6_-tag (pink) and a SUMO-domain (blue) followed by the 17 N-terminal amino acids of Huntingtin (N17, orange). The different Poly-Q encoding sequences (red) and a FLAG-tag (yellow) are located at the 3' end. Expression is controlled by a T7 promoter (brown). For cloning of the final constructs the Poly-Q encoding repeats of different lengths were excised from of the pMK1-Q_n _vectors using BsaI and BsmBI. The resulting fragments were inserted into pLANA, which was linearized with BsaI. The restriction sites were arranged in a way that allowed the seamless ligation of the Poly-Q encoding sequences behind N17 into pLANA. Our final constructs still contain a BsaI site behind the Poly-Q encoding region allowing further nucleotide insertions. Used restriction sites are underlined. **(B**) Three different constructs were successfully produced in *E. coli *and purified. The proteins consisting of His_6_-SUMO-N17-Q_n_-FLAG were applied to SDS-PAGE and visualized by immunoblotting against the FLAG-tag. **(C) **Purified His_6_-SUMO-N17-Q_47_-FLAG fusion proteins were treated with Ulp1 for 1 min and analyzed by SDS-PAGE and α-FLAG immunoblotting. **(D) **Filter retardation assay using His_6_-SUMO-N17-Q_47_-FLAG fusion proteins. Membrane bound Poly-Q fibrils were visualized by α-FLAG immunodetection.

Our initial intention was to generate Poly-Q constructs for *in vitro *experiments. So far, we cloned repetitive DNA sequences of different lengths encoding Poly-Q stretches between 11 and 218 residues into the pMK1 vector (Figure 3A). We next adopted these constructs to meet our specific purposes. In our final constructs the 17 N-terminal amino acids of Htt (N17) flank the different Poly-Q regions and a FLAG-tag for immunodetection is located at the C-terminal end. In order to insert the various Q-blocks between the N17 and FLAG-tag encoding sequences on the expression vector (pLANA), the plasmid was linearized with the BsaI enzyme. The glutamine encoding sequences were excised from the pMK1 plasmids with BsaI and BsmBI resulting in compatible ends that allowed the seamless insertion of Q-blocks of different lengths behind the N17 motif (Figure 6A).

To study the aggregation kinetics of Poly-Q proteins *in vitro*, we applied a widely used filter retardation assay, which allows the detection and quantification of small amounts of Poly-Q containing aggregates [5]. This assay is based on the characteristic of Poly-Q fibrils to be insoluble in solutions containing 2% sodium dodecyl sulfate (SDS). Such fibrils are specifically retained on a cellulose-acetate filter, whereas the soluble monomeric species are denatured by SDS and filtered through the membrane. The captured aggregates can then be visualized by immunodetection. For such experiments fusion constructs were commonly used carrying a N-terminal glutathione *S*-transferase (GST) domain fused to the first exon of *htt *via a factor Xa cleavage site [5]. In these constructs the globular GST domain prevents aggregation of the monomers. However, aggregation can be induced by release of the GST moiety upon proteolytic factor Xa cleavage. Based on this principle we designed a new construct in which the first exon of the *htt *gene was fused to a SUMO domain (*Saccharomyces cerevisiae *Smt3) carrying an N-terminal His_6_-tag for affinity purification (Figure 6A). Importantly, the His_6_-SUMO domain keeps the purified Poly-Q containing fusion proteins soluble and allows for the controlled induction of aggregation by treatment with the Ulp1 protease (SUMO protease from *S. cerevisiae*), which cleaves behind the two conserved C-terminal glycine residues of folded SUMO [21].

Using the His_6_-SUMO fusion strategy the Poly-Q proteins can be easily affinity purified under denaturing conditions using silica-based Ni^2+^-matrices (Figure 6B). As Poly-Q proteins often tend to form insoluble inclusions when expressed in *E. coli*, we purified our constructs under denaturing conditions using 6 M guanidine hydrochloride and refolded them on the affinity matrix by slowly decreasing the concentration of the denaturant. The efficiency of the refolding process was monitored by digestion of the purified and soluble Poly-Q fusion proteins with Ulp1 (Figure 6C). Importantly, this protease does not recognize a specific amino acid sequence but rather the native structure of the SUMO-domain. Under our experimental conditions the purified fusion proteins were almost completely digested by Ulp1 within one minute, indicating that refolding of the constructs was successful. Therefore, we performed the filter retardation assay with the Poly-Q fusion proteins and induced aggregation by Ulp1 mediated cleavage. Figure 6D exemplifies the assay with our construct containing 47 glutamine residues. In the control reaction, where Ulp1 was omitted, no aggregation was observed over six hours beyond marginal background signals. By contrast, significant amounts of SDS-resistant Poly-Q fibrils were formed after one hour upon induction of SUMO cleavage. Accordingly, release of His_6_-SUMO from the Poly-Q constructs occurs much faster than fibril formation (Figure 6C and 6D). Thus, our His_6_-SUMO fusion strategy provides a useful tool to study the kinetics of Poly-Q aggregation *in vitro*.

## Conclusion

As mentioned above, several other seamless cloning strategies were described previously, indicating the demand for such methods. However, our approach has several advantages compared to existing methods. Here we used synthetic oligonucleotides to overcome the limitations of PCR-based amplification of repetitive DNA sequences and combined this strategy with seamless cloning using Type IIS restriction enzymes. The usefulness of this method was demonstrated by means of two frequently occurring problems: the cloning of defined stretches of Poly-Q encoding sequences and the generation of polyadenine sequences for *in vitro *transcription of polyadenylated mRNA. We chose the latter application because we wanted to demonstrate that the technique is suitable for generating fully homogenous nucleotide sequences of defined length. Another important advantage is that our method is directed. This means that in each elongation cycle, the number of nucleotides added to the repetitive sequence can be determined precisely by simply designing the oligonucleotides accordingly. Importantly, a single product is obtained in each step. Other methods often generate a mixture of products due to multiple insertions. Consequently, products of the right length or sequence have to be selected or purified via agarose gels, which makes the procedure more complicated, time-consuming, and error-prone. As we did not observe multiple insertions and only rarely religations of the vector, this is not necessary using our technique. In addition, the protocol presented here is straightforward. One round of elongation can be done within one day, including restriction digestion of the vector, ligation, and transformation. Bacterial growth and sequencing requires approximately two further days. We obtained approximately 100-300 clones per transformation and in most cases it was sufficient to sequence a single clone. Moreover, combining pre-existing constructs can rapidly increase the length of the repetitive sequence (Figure 2 and 3). For example, once the method was established, the pMK1-constructs could be generated within three weeks including sequencing (Figure 3A). We did not encounter a limitation of the number of repeats for our purposes.

In general, our method is cheap because only the synthetic oligonucleotides have to be purchased for each application. We usually ordered 0.02 μmol of commercially synthesized, HPLC-purified oligonucleotides, respectively. The costs depend therefore on the list price per nucleotide, purity, scale, and length of the oligonucleotides. Once annealed and digested, the oligonucleotides can be used for all subsequent elongation rounds and do not have to be re-ordered. All other materials (e.g. restriction enzymes, agarose gels, competent cells, DNA ligase) are standard equipment for molecular cloning. It should be mentioned that in principle the strategy could be used also to assemble non-repetitive sequences. In this case, the DNA fragments could be amplified even by PCR with primers carrying the Type IIS and Type IIP restriction sites in the 5' overhang regions. Finally, it should be also possible to fuse different repetitive sequences in a directed and seamless manner (Figure 5).

In summary, our seamless cloning strategy is PCR-free and allows the directed and efficient generation of highly repetitive DNA sequences of defined lengths by simple standard cloning techniques. Besides their applications in basic research, as exemplified here, repetitive nucleotide sequences become increasingly important in biopolymer technology. As artificial proteins with unique physical properties such as elastomeric polypeptides and synthetic silk fibers often contain multiple amino acid repeats, new strategies are required that improve the cloning of synthetic genes for the production of protein-based polymeric materials in biological systems [22,23]. The method presented here is both, cheap and fast, and can be easily adapted to produce any desired DNA sequences for a wide range of applications.

## Methods

### Cloning Methods

All cloning steps were performed according to standard protocols [24]. Restriction enzymes were named according to [25]. HPLC-purified synthetic Q-block (5'-ccagtgGGTCTCaCAGCAACAGCAACAGCAGCAGCAACAGCAGcagagacgGAG CTCgatc-3' and 5'-gatcGAGCTCcgtctctgCTGCTGTTGCTGCTGCTGTTGCTGTT GCTGtGAGACCcactgg-3') and poly(A) oligonucleotides A_31 _(5'-gccgAAGCTT aaaaaaaaaaaaaaaaaaaaaaaaaaaaaaaGAGACCcactgg-3' and 5'-ccagtgGGTCTCtttttttttttttttttttttttttt tttttAAGCTTcggc-3') and A_32 _(5'-ccagtgGGTCTCAaaaaaaaaaaaaaaaaaaaaaaaaaaaaaaAGAGACG acagCTCGAGgccg-3' and 5'-cggcCTCGAGctgtCGTCTCTttttttttttttttttttttttttttttttTGAGACCcactgg-3') were obtained from Thermo Fisher Scientific. For annealing 10 pmol of the antiparallel oligonucleotides were mixed in a final volume of 100 μl. The reaction mixture was heated up to 95°C for 5 min, incubated for 10 min at 55°C, and finally cooled down to room temperature. The annealed oligonucleotides were digested with the respective restriction endonucleases (NEB) according to the manufacturers protocol. Next, the DNA was precipitated by addition of 1/10 volume 3 M sodium acetate pH 5.2, 2.5 volumes of 100% (v/v) ethanol (p.a.) and incubation at -20°C for at least 12 h. The DNA was recovered by centrifugation at 16000 × g for 30 min at 4°C and the DNA pellets were washed with 70% (v/v) ethanol. The DNA pellets were dried at room temperature and subsequently resuspended in 100 μl sterile water. Aliquots of the double-stranded digested oligonucleotides were stored at -20°C and used for the subsequent cloning cycles to elongate the repetitive sequences. Between 0.1 μl and 5 μl of the digested oligonucleotides were ligated with 30-70 ng linearized and dephosphorylated plasmid DNA and T4 DNA ligase (Fermentas) in a 20 μl reaction mixture according the manufacturers instructions. The ligations were carried out at 23°C for 2 h and transformed into 100 μl RbCl_2_-competent *E. coli *cells (strain: DH5αZ1). Plasmid preparation, gel extraction, and DNA purification kits were obtained from Qiagen. All DNA fragments originating from plasmid DNA were separated by agarose gel electrophoresis and extracted using the QIAquick Gel Extraction Kit (Qiagen). All plasmids generated during this study were verified by DNA sequencing (GATC, Germany).

To generate the poly(A) constructs the protocol was adapted due to the lower melting temperature of the poly(A) oligonucleotides. The antiparallel oligonucleotides were mixed as described above, heated to 95°C, and annealed by a continuous temperature gradient of 0.1°C per second to 4°C. To avoid any denaturation of poly(A) stretches the annealed oligonucleotides as well as vectors containing poly(A) regions were digested with the Type IIS restriction enzymes overnight at 37°C. Restriction enzymes were removed by phenol/chloroform extraction according to standard protocols and the DNA was recovered by ethanol precipitation as described above. The resulting poly(A) constructs were analyzed by restriction digestions and the lengths of the poly(A) stretches up to 100 base pairs could be determined precisely by sequencing (GATC, Germany).

### Expression and purification of Poly-Q proteins

The pLANA-Q_n _constructs (Figure 6A) were transformed into the *E. coli *strain BL21(DE3). A two liter LB-culture was inoculated with 20 ml of a stationary overnight culture and grown to an OD_600 nm _of 0.6-0.8 at 30°C. Expression was induced by the addition of 1 mM isopropylthio β-D-1-galactopyranoside (IPTG). After 5 h cells were harvested by centrifugation and flash-frozen in liquid nitrogen. The cell pellets were resuspended in lysis buffer (30 mM HEPES-KOH pH7.4, 500 mM potassium acetate, 5 mM magnesium chloride 5% (v/v) glycerol, 1 mM ß-mercaptoethanol, 1 mM phenylmethylsulphonyl fluoride (PMSF), 1 × protease inhibitor cocktail (Roche)) and lysed by French press. After centrifugation at 30000 × g for 30 min at 4°C the pellet was resuspended in denaturation buffer 1 (6 M guanidine hydrochloride (GdnHCl), 30 mM HEPES-KOH pH 7.4) and incubated with 2.5 g Ni-IDA silica matrix (Protino; Macherey-Nagel) for 30 min at 4°C while rotating. The matrix was washed twice with 40 ml denaturation buffer 1, twice with 40 ml denaturation buffer 2 (4 M GdnHCl, 30 mM HEPES-KOH pH 7.4), twice with 40 ml denaturation buffer 3 (2 M GdnHCl, 30 mM HEPES-KOH pH 7.4), twice with 40 ml high salt buffer (30 mM HEPES-KOH pH 7.4, 1 M potassium acetate, 5 mM magnesium chloride, 5% (v/v) glycerol, 1 mM ß-mercaptoethanol), and finally four times with with 40 ml low salt buffer (30 mM HEPES-KOH pH 7.4, 50 mM potassium acetate, 5 mM magnesium chloride, 5% (v/v) glycerol, 1 mM ß-mercaptoethanol). Proteins were eluted with four times 10 ml elution buffer (30 mM HEPES-KOH pH 7,4, 50 mM potassium acetate, 5 mM magnesium chloride, 5% (v/v) glycerol, 1 mM ß-mercaptoethanol, 250 mM imidazole-HCl pH 8.0). The purest fractions were dialyzed against 5 l low salt buffer for 3 h. To avoid aggregation the purified proteins were diluted, frozen in liquid nitrogen, and stored at -80°C. The purified proteins were analyzed by SDS-PAGE and Coomassie staining as well as immunoblotting using FLAG antibodies (ANTI-FLAG, Sigma).

### Filter retardation assay

The Q-containing fusion proteins were thawed on ice and diluted with low salt buffer (30 mM HEPES-KOH pH 7.4, 50 mM potassium acetate, 5 mM magnesium chloride, 1 mM ß- mercaptoethanol, 10% (v/v) glycerol) to a final concentration of 0.02 mg/ml. Insoluble material was removed by centrifugation at 16000 × g for 5 min at 4°C. The supernatant was divided into two samples, which were directly used for the filter retardation assay. The SUMO protease was added to one of the samples to a final concentration of 10 μg per mg substrate protein. The control reaction contained BSA instead of protease. The reactions were incubated at 22°C in a final volume of 350 μl. At the indicated time points 50 μl samples (1 μg fusion protein) were taken and mixed 1:1 with stop solution (4% (w/v) SDS, 100 mM dithiothreitol) followed by incubation at 95°C for 5 min. Under these conditions monomeric Poly-Q proteins are denatured whereas Poly-Q aggregates remain intact. Subsequently, the samples were applied to a Dot-blot filtration unit and filtered through a cellulose-acetate membrane (0.2 μm pore size) equilibrated with 2% (w/v) SDS in TBS (100 mM Tris-HCl pH 8.0, 150 mM sodium chloride). After washing with 0.1% (w/v) SDS in TBS the membrane was blocked in TBS-T (100 mM Tris-HCl pH 8.0, 150 mM sodium chloride, 0.05% (v/v) Tween-20) containing 5% (w/v) non-fat dried milk. The aggregates were detected by immunoblotting using primary FLAG-antibodies and alkaline-phosphatase coupled to anti-rabbit IgG (1:10000, Sigma).

## Authors' contributions

AS cloned the Poly-Q and poly(A) constructs, performed *in vitro *aggregation assays, and wrote the paper; SP developed the cloning strategy, designed the oligonucleotides, contributed in cloning of the poly(A) constructs, and wrote the paper; MK performed site-directed mutagenesis and contributed in cloning of the poly(A) constructs; ED coordinated the project and assisted in manuscript writing. All authors read and approved the final manuscript.
